# Predicting Factors Associated With Uncontrolled Hypertension Using Machine Learning Methods: A Cross-Sectional Study in Western Iran

**DOI:** 10.1155/ijhy/4011397

**Published:** 2025-02-18

**Authors:** Zahra Cheraghi, Mahboobeh Doosti-Irani, Masoumeh Sohrabi, Amin Doosti-Irani

**Affiliations:** ^1^Department of Epidemiology, School of Public Health, Modeling of Noncommunicable Diseases Research Center, Institute of Health Sciences and Technologies, Hamadan University of Medical Sciences, Hamadan, Iran; ^2^Department of Epidemiology, School of Public Health, Hamadan University of Medical Sciences, Hamadan, Iran; ^3^Department of Mathematical Sciences, Isfahan University of Technology, Esfahan, Iran; ^4^Department of Epidemiology, School of Public Health, Research Center for Health Sciences, Institute of Health Sciences and Technologies, Hamadan University of Medical Sciences, Hamadan, Iran

**Keywords:** cross-sectional studies, health literacy, Iran, uncontrolled blood pressure

## Abstract

Uncontrolled hypertension is a major public health issue globally. This study aimed to uncover the factors contributing to uncontrolled hypertension using machine learning techniques. In this study, 303 adults with hypertension were included in this cross-sectional study. Data were collected using the Standard Health Literacy Questionnaire. Uncontrolled hypertension was defined as systolic blood pressure (BP) ≥ 140 mmHg and/or diastolic BP ≥ 90 mmHg on both days. Data were analyzed using percentages and chi-square tests. Four machine learning algorithms were employed in this study. The efficacy of these algorithms was assessed using several performance metrics, including accuracy, positive predictive value, sensitivity, *F*_Score, and the area under the receiver operating characteristic (ROC) curve (AUC). The analyses were performed utilizing Python version 3.8. Of the four models evaluated, logistic regression exhibited the highest accuracy at 75.4% and the greatest AUC at 0.87. According to the logistic regression algorithm, individuals who did not adhere to their treatment had a significantly lower likelihood of having uncontrolled hypertension (OR = 0.17, *p* value < 0.001). Number of children (OR = 0.44, *p* < 0.001), physical activity (OR = 0.94, *p* < 0.001), and health literacy (OR = 0.29, *p* = 0.10) were all associated directly, and salt intake (OR = 9.60, *p* < 0.001) was associated inversely with the odds of having uncontrolled hypertension. Based on variable importance analysis, low physical activity was identified as the most important variable, followed by weak health literacy and nonadherence to drug treatment. Factors such as age, duration of hypertension, chronic disease, and salt consumption were also significant. Adherence to treatment, physical activity, health literacy, and salt intake play crucial roles in uncontrolled hypertension. Interventions targeting these factors could help in managing and preventing uncontrolled hypertension.

## 1. Introduction

Hypertension is a significant public health concern in many communities due to its association with premature deaths worldwide. Uncontrolled hypertension is defined as a condition where systolic blood pressure (BP) is ≥ 140 mm·Hg or diastolic BP is ≥ 90 mm·Hg, despite medical therapy, after two recent medical visits. The primary cause of the increased prevalence of patients who cannot control their BP is poor hypertension control, including noncompliance with treatment [1–3].

According to the World Health Organization (WHO) report, around 4 in 5 people with high BP are not adequately treated, but if countries can scale up coverage, 76 million deaths could be prevented between 2023 and 2050 [4]. The prevalence of hypertension, defined as a BP of 140/90 mmHg or higher or taking medication for hypertension, has doubled from 650 million to 1.3 billion between 1990 and 2019. Currently, almost half of the global population with hypertension are unaware of their condition. Furthermore, more than three-quarters of adults with hypertension reside in low- and middle-income countries [5].

According to the WHO, in 2008, an estimated 30.7% of men and 29.1% of women in the Eastern Mediterranean Region had hypertension. In the Islamic Republic of Iran, the prevalence of hypertension among adults aged over 25 years was estimated to be 31% in men and 27% in women [6].

Previous studies have identified several independent predictors of uncontrolled hypertension, including nonadherence to antihypertensive drugs, being overweight, nonadherence to physical exercise, and nonadherence to alcohol withdrawal [6]. Additionally, uncontrolled hypertension was found to be more prevalent among single individuals, families with low income, and those with albuminuria or decreased renal function, as well as those with lower adherence to antihypertensive drugs [6]. The objective of this study is to identify the factors contributing to the uncontrolled hypertension among adults in the Islamic Republic of Iran, with a focus on exploring the prevalence of nonadherence to antihypertensive medications, lifestyle choices, and socioeconomic status. This research aims to provide insights that could inform public health strategies to improve hypertension management and reduce the burden of this condition in the region.

## 2. Materials and Methods

This analytical cross-sectional study analyzed a sample of patients previously diagnosed with hypertension by a physician and who had a medical record in health centers in Hamadan city (west of Iran) during the second half of 2019. The patients were selected through stratified random sampling from twenty-four comprehensive urban health service centers (CURHCs) in Hamadan city. A total of 10 out of the 25 available centers were selected through a simple random method, which was determined using a random number table. The study's inclusion criteria required participants to have hypertension, be over 18 years old, and use antihypertensive drugs. Individuals who were pregnant or elderly with Alzheimer's disease were excluded from the study.

The study by Arabzadeh et al. [2] was used to calculate the sample size, which reported a prevalence of uncontrolled hypertension at 62.28%. The maximum acceptable error was set at 0.12 with a 95% confidence level.

Uncontrolled hypertension was ascertained based on the WHO definition in this study. Uncontrolled hypertension is defined as a condition where, after at least two recent medical visits and despite medical therapy, systolic BP is ≥ 140 mm·Hg or diastolic BP is ≥ 90 mm·Hg. The use of antihypertensive medication was self-reported, and BP status was measured by the physician during periodic care [7].

The following data were collected for both groups of patients, those with controlled hypertension and those with uncontrolled hypertension: demographic information, smoking history (past/current or none), alcohol consumption (yes or no), physical activity (hours per week), adherence to antihypertensive medication (yes or no), and a history of hypertension in first-degree family members (yes or no).

Four algorithms were utilized to identify the underlying factors contributing to uncontrolled hypertension: decision tree, random forest, support vector machine (SVM), and logistic regression. The decision tree algorithm is characterized by its interpretability, enabling healthcare professionals to comprehend and articulate the rationale behind clinical diagnoses. This attribute is particularly significant within the medical domain, where the processes of reasoning and interpretation are paramount. To mitigate the issue of overfitting inherent in the decision tree model, we implemented various parameters, including the number of trees, their depth, and the selection of the criterion index. This approach facilitated the determination of the optimal selection between entropy and Gini impurity, thereby enhancing the model's robustness. Random forests are a robust method for handling complex datasets, even when they contain missing or corrupted data. They provide a measure of feature importance, which allows for the identification of relevant variables in medical diagnosis [8]. Additionally, random forests can capture nonlinear relationships between variables, which is crucial for understanding complex medical conditions [9]. In order to tackle the problem of overfitting in random forest algorithm, hyperparameter tuning was performed, including tuning the number and depth of trees, as well as the desired number of features. In addition, techniques such as cross-validation and regularization were used.

In summary, SVM is well-suited for handling high-dimensional medical data and can effectively separate different classes. SVMs can capture complex relationships and improve classification accuracy using nonlinear kernels. SVM's ability to generalize and its robustness to noisy data make it suitable for medical diagnostics. Its success in medical research contributes to its popularity in this field [10].

The SVM has three tuning parameters that should be optimized. The parameters of the SVM in this kernel function were tuned using trial and error. To do this, we used 10-fold cross-validation technique in the training dataset. SVM is a supervised machine learning algorithm used for classification and regression. In this study, in order to address the issue of imbalanced data, we utilized hyperparameters in the SVM model. The SVM model was constructed using a radial basis function (rbf) as the kernel, in addition to the gamma and C parameters. The gamma parameter indicates the impact of a single training data instance on the decision boundary or hyperplane. C, on the other hand, controls the balance between accurately classifying training examples and maximizing the margin of the decision function. The C parameter serves as a regularization parameter in SVM, aiding in the prevention of overfitting. Specifically, the C parameter in this study encompassed the values [0.001, 0.01, 0.1, 1, 10, 100]. Logistic regression is also a suitable choice for medical diagnosis as it provides interpretable coefficients, making it easier for medical professionals to understand the relationship between input variables and outcomes.

Logistic regression is a useful tool in medical diagnosis as it can handle both categorical and continuous input variables, allowing for the inclusion of various types of data.

It is efficient with small datasets, which is often the case in medical diagnosis where data collection can be costly and complex. Additionally, the model stability of logistic regression is advantageous as it requires fewer assumptions compared to other classification methods, making it more robust in different situations [11].

To apply the four models, the data for each county were divided into two subsets. 80% of the data was utilized as the training set, while the remaining 20% was designated as the test set. Various techniques were employed to enhance the reliability of the predictive analysis through cross-validation. These techniques included training the model on different subsets of data to ensure generalizability, utilizing holdout validation to evaluate performance on unseen data, implementing stratified sampling to ensure representative class distributions, selecting features to improve performance and interpretability, tuning hyperparameters to optimize model settings, utilizing ensembling to combine models for improved results, conducting error analysis to identify areas for improvement, and performing sensitivity analysis to test model robustness. These techniques are crucial in the development of accurate and dependable machine learning models.

The performance of algorithms was assessed using the following measures: accuracy, precision, sensitivity, *F*_Score, and area under the receiver operating characteristic (ROC) curve (AUC) in ROC analysis. The AUC is a measure of the algorithm's ability to distinguish between hypertensive and normotensive individuals. It is calculated as the area under the curve when plotting true positive rates against false positive rates using different cutoff values. The calculation formula is as follows:

(1) $$\begin{array}{c} \text{Accuracy}=\frac{\text{True Positive}+\text{True Nagative}}{\text{Total }},\\ \text{Sensitivity}=\frac{\text{True Positive}}{\text{True Positive}+\text{False Nagative}},\\ F-\text{Measure}:\frac{2}{\left(1/\text{Positive Predictive Value}\right)}+\frac{1}{\text{Sensitivity}}. \end{array}$$

The variable importance criterion assesses the influence of covariates on the outcome variable. This criterion relies exclusively on the role of predictors in building a tree. In this study, variable importance was calculated using the decrease in the impurity method, which evaluates the average reduction in impurity (measured by the Gini index) due to splits on a specific variable across all trees in the forest. A greater reduction in impurity indicates a higher importance of the variable. All analyses were conducted using Python 3.8.

The Institutional Review Board (IRB) of the ethics committee of the Hamadan University of Medical Sciences approved the study protocol (IR.UMSHA.REC.1402.615). Informed consent was obtained and confirmed by the IRB.

## 3. Results

This cross-sectional study was conducted on 303 adults with hypertension, of which 153 (50.4%) had uncontrolled hypertension. The uncontrolled hypertension group had a higher average age compared to the controlled hypertension group (59.3 years vs. 50.6 years, *p* < 0.001). There was a statistically significant difference in the gender distribution between the two groups (*p* = 0.049), with a higher percentage of males in the uncontrolled hypertension group. Marital status showed no significant difference between the two groups (*p* = 0.120). The uncontrolled hypertension group had a significantly higher number of children compared to the controlled hypertension group (3.9 children vs. 3.3 children, *p* = 0.001). More details are provided in Table 1.

Based on the logistic regression algorithm, individuals who did not adhere to their treatment had an adjusted odds ratio of 0.17, indicating a significantly lower likelihood of having uncontrolled hypertension (*p* value < 0.001, 95% CI: [0.07, 0.42]). Each additional year of age was associated with 1.05 times increased odds of having uncontrolled hypertension, although this result was marginally significant (*p* value = 0.071, 95% CI: [1.00, 1.11]).

For each additional child, the odds of having uncontrolled hypertension decreased by 0.44 times (*p* value < 0.001, 95% CI: [0.30, 0.65]).

Individuals with chronic diseases had an adjusted odds ratio of 2.36, suggesting a higher likelihood of having uncontrolled hypertension, although this result was marginally significant (*p* value = 0.058, 95% CI: [0.97, 5.74]).

Each additional salt package purchased per month was associated with 9.60 times increased odds of having uncontrolled hypertension (*p* value < 0.001, 95% CI: [3.19, 28.9]).

For every additional hour of physical activity per week, the odds of having uncontrolled hypertension decreased by 0.94 times (*p* value < 0.001, 95% CI: [0.92, 0.97]).

Each additional score in health literacy was associated with decreased odds of having uncontrolled hypertension (adjusted odds ratio = 0.97, *p* value = 0.010, 95% CI: [0.95, 0.99]).

The frequency of check-up did not have a significant impact on uncontrolled hypertension (adjusted odds ratio = 0.29, *p* value = 0.289, 95% CI: [0.26, 1.50]) (Table 2).

**Figure 1:**
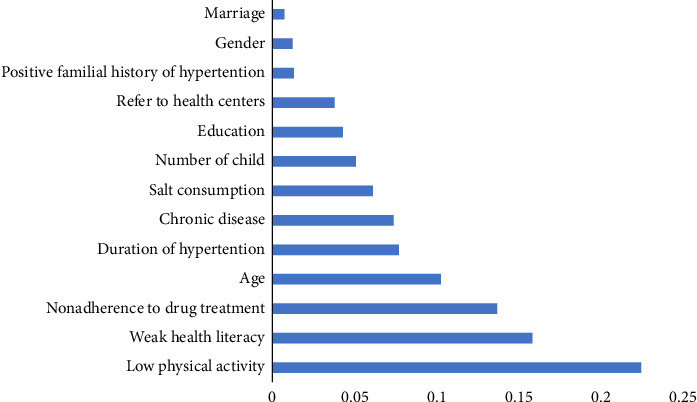
Relative importance of predictors of uncontrolled hypertension.

According to the variable importance analysis, the variable “Low Physical Activity” has the highest importance with a value of 0.22. The variables “Weak Health Literacy” and “Nonadherence to Drug Treatment” have moderate importance with values of 0.15 and 0.13, respectively. The variables “Age” and “Duration of Hypertension” also have some importance with values of 0.10 and 0.08, respectively. The variables “Chronic Disease” and “Salt Consumption” have lower importance with values of 0.07 and 0.06, respectively. The variables “Number of Child,” “Education,” “Refer to Health Centers,” “Positive Familial History of Hypertension,” “Gender,” and “Marriage” have the least importance with values ranging from 0.01 to 0.01. Overall, these results suggest that low physical activity, weak health literacy, and nonadherence to drug treatment are the most significant factors in predicting the outcome. Other variables, such as age, duration of hypertension, chronic disease, and salt consumption, also play a role but to a lesser extent. The remaining variables have a minimal impact on the outcome (Figure 1).

**Figure 2:**
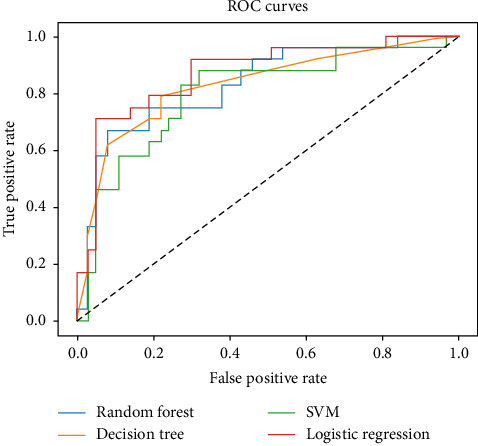
Comparing the area under the curve in four types of machine learning algorithms.

According to the results shown in Table 3, the machine learning algorithms demonstrated varying levels of performance on the test data for positive values. From the results for the different models, it is clear that logistic regression achieved the highest accuracy at 75.4% and the highest AUC of 0.87 among the models tested. In contrast, the decision tree model had the highest precision at 82.0, while the SVM model showed the highest sensitivity at 70.8. Logistic regression also excelled in *F*_Score, scoring 0.72. Overall, all models reported an AUC of approximately 0.83, with random forest having the lowest *F*_Score of 61.0 (see Table 3 and Figure 2).

## 4. Discussion

In this study, we aimed to identify the predictors of uncontrolled hypertension. We conducted a variable importance analysis to determine the relative importance of various factors in predicting the outcome. The results showed that low physical activity, weak health literacy, and nonadherence to drug treatment were the most important factors for uncontrolled hypertension.

Regular physical activity is important for managing and controlling hypertension. It can lower BP, improve cardiovascular health, help with weight management, reduce insulin resistance, and alleviate stress. These benefits make physical activity an effective and essential component of hypertension management and prevention [12]. Also, due to reduced physical activity in the elderly, the problem of hypertension control is impaired. In the present study, and according to the multivariate analysis in the present study, with increasing hours of physical activity duration, the probability of uncontrolled hypertension in patients declined significantly by 6% (OR = 0.96). Similar studies have found an inverse relationship between physical activity and uncontrolled hypertension [13, 14].

Health literacy was the second important variable. It can influence hypertension control in various ways, such as challenges in understanding the condition, following medication regimens, making lifestyle changes, and communicating effectively with healthcare providers. Those with low health literacy may find it difficult to appreciate the importance of hypertension management, often leading to inadequate adherence to treatment plans and lifestyle suggestions. To bolster health literacy concerning hypertension control, healthcare providers can utilize clear language, furnish accessible educational materials, and motivate patients to inquire about their health. By tackling health literacy obstacles, individuals can obtain a clearer understanding of their conditions and attain better BP management. Several studies have indicated that the aspect of health literacy, regardless of other factors, is significant in adherence to the treatment of various diseases [15–17]. Moreover, in this research, the health literacy score was higher among patients who adhered to treatment (64.8 vs. 46.3). There was also a significant inverse association between each additional point in health literacy and the odds of uncontrolled hypertension, suggesting that adherence to treatment is effective in helping patients achieve better control of their condition.

The third important variable was and nonadherence to drug treatment. Nonadherence to drug treatment for hypertension can lead to poor BP control and increased risk of complications. Qualitative studies showed that reasons for nonadherence include forgetfulness, side effects, financial constraints, lack of understanding, or feeling that medication is unnecessary [18, 19]. Healthcare providers should address barriers to adherence, provide education, simplify regimens, and offer support. Open communication between patients and providers is key to improving adherence and achieving better health outcomes. Also, based on the logistic regression algorithm, individuals who did not adhere to their treatment had an adjusted odds ratio of 0.17, indicating a significantly lower likelihood of having uncontrolled hypertension that was significant association.

Age, duration of hypertension, chronic diseases, and salt consumption were found to contribute to the risk of uncontrolled hypertension, albeit to a lesser degree. Additional factors such as the number of children, education level, frequency of visits to health centers, family history of hypertension, gender, and marital status had minimal impact on the outcomes. These findings offer valuable insights for healthcare providers and policymakers, aiding in the development of interventions aimed at improving BP control and mitigating the burden of uncontrolled hypertension.

### 4.1. Limitations and Strengths

The main objective of this study was to investigate the occurrence of uncontrolled hypertension, a chronic and potentially harmful condition that can lead to other preventable illnesses. However, it is important to note that one of the significant limitations of this study is its cross-sectional design, which means that the identified associations may not necessarily imply causation. Consequently, the necessity for future longitudinal studies (cohort studies) employing a more robust statistical approach is apparent. This enhanced statistical approach should account for potential confounders and provide more insights into the directionality of the relationships.

## 5. Conclusions

Given the findings, it is evident that factors such as low physical activity, poor health literacy, and nonadherence to drug treatment play crucial roles in the development of uncontrolled hypertension. This underscores the importance of promoting regular physical activity, enhancing health literacy, and addressing obstacles to medication adherence in both the management and prevention of hypertension. As such, healthcare providers should prioritize patient education, offer easily understandable materials, and foster open communication to enhance health outcomes for individuals with hypertension. This highlights the significance of translating research into practical recommendations for clinical practice and public health policy.

## Figures and Tables

**Table 1:** Baseline characteristics of participants.

Variables	Uncontrolled hypertension (*N* = 153)	Controlled hypertension (*N* = 150)	*p* value
Age (years)	59.3 ± 12.7	50.6 ± 11.6	< 0.001
Gender (%)			0.049
Male	45.8	34.7	
Female	54.2	65.3	
Marital status (%)			0.120
Married	85.6	91.3	
Single/divorced/widowed	14.4	8.7	
Number of children	3.9 ± 1.7	3.3 ± 1.9	0.001
Income (monthly mean in million)	1.8 ± 0.9	2.3 ± 1.1	< 0.001
Smoking (%)			0.001
Current/past	22.9	8.7	
Education level (%)			< 0.001
Higher than diploma	3.9	14.7	
Diploma/below diploma	15.0	19.3	
Primary school	36.6	44.0	
Illiterate	44.4	22.0	
^†^FHH (%)	52.9	50.7	
Physical activity (times in a week)	2.1 ± 1.8	3.6 ± 1.4	< 0.001
^††^SPP	1.1 ± 0.6	0.8 ± 0.3	< 0.001
Health literacy score (mean)	46.3 ± 22.2	64.8 ± 23.7	< 0.001
Frequency of check-up (%)			
4 times per year	31.4	38.00	0.003
6 times per year	21.00	14.00	
12 times per year	22.2	35.3	
24 times per year	25.5	12.7	

*Note:* Unpaired *t*-test and analysis of variance (ANOVA) for quantitative variables and chi-squared test for qualitative variables were used.

^†^Familial history of hypertension.

^††^Number of salt packages purchased per month.

**Table 2:** The role of related factors on uncontrolled hypertension according to the logistic regression algorithm (based on training dataset (80%) *n* = 242).

Variables	Adjusted odds ratio	*p* value	95% CI
Adherence of treatment (yes/no)	0.17	< 0.001	[0.07, 0.42]
Age (year)	1.05	0.071	[1.00, 1.11]
Child number (per one)	0.44	< 0.001	[0.30, 0.65]
Chronic diseases (yes/no)	2.36	0.058	[0.97, 5.74]
NSPP^†^ (per one)	9.60	< 0.001	[3.19, 28.9]
Physical activity (hours in week)	0.94	< 0.001	[0.92, 0.97]
Health literacy (per one score)	0.97	0.010	[0.95, 0.99]
Frequency of check-up (per one time in month)	0.29	0.289	[0.26, 1.50]

^†^Number of salt packages purchased per month.

**Table 3:** Performance of the machine learning algorithms on test data for positive value.

Model	Accuracy	Precision	Sensitivity	*F*_Score	AUC
Logistic regression	75.4 (68.9, 89.4)	0.66 (54.1, 77.9)	84.38 (57.8, 92.9)	0.72 (60.7, 83.3)	0.87 (78.6, 95.4)
Decision tree	75.0 (65, 3, 84.7)	82.0 (73.4, 90.6)	69.0 (58.7, 79.4)	75.0 (65, 3, 84.7)	83.0 (69.9, 90.0)
Support vector machine	72.0 (60.7, 83.3)	63.0 (50.8, 70.1)	70.8 (59.4, 82.2)	67.0 (55.2, 78.8)	80.0 (69.9, 90.0)
Random forest	69.0 (58.7, 79.4)	62.0 (49.8, 74.1)	70.8 (59.4, 82.2)	61.0 (48.8, 73.2)	83.0 (69.9, 90.0)

## Data Availability

The raw data that were utilized in this study are available upon request from the corresponding author.

## Nomenclature

BP: Blood pressure
WHO: World Health Organization
CURHCs: Comprehensive urban health service centers
SVM: Support vector machine
rbf: Radial basis function
AUC: Area under the ROC curve.

## References

[1] Cho J., Kim C., Kang D. R., Park J. B. (2016). Hyperuricemia and Uncontrolled Hypertension in Treated Hypertensive Patients: K-MetS Study. *Medicine*.

[2] Arabzadeh S., Sadeghi M., Rabiei K., Sarrafzadegan N., Taheri L., Golshahi J. (2014). Determinants of Uncontrolled Hypertension in an Iranian Population. *ARYA atherosclerosis*.

[3] Abd El-Aty M. A., Meky F. A., Morsi M. M., Al-Lawati J. A., El Sayed M. K. (2015). Hypertension in the Adult Omani Population: Predictors for Unawareness and Uncontrolled Hypertension. *Journal of the Egyptian Public Health Association*.

[4] WHO First WHO Report Details Devastating Impact of Hypertension and Ways to Stop It. https://www.who.int/news/item/19-09-2023-first-who-report-details-devastating-impact-of-hypertension-and-ways-to-stop-it.

[5] Kearney P. M., Whelton M., Reynolds K., Whelton P. K., He J. (2004). Worldwide Prevalence of Hypertension: A Systematic Review. *Journal of Hypertension*.

[6] Katibeh M., Moghaddam A. S., Yaseri M., Neupane D., Kallestrup P., Ahmadieh H. (2020). Hypertension and Associated Factors in the Islamic Republic of Iran: A Population-Based Study. *Eastern Mediterranean Health Journal*.

[7] WHO (2022). Hypertention. https://www.who.int/health-topics/hypertension.

[8] Azar A. T., El-Metwally S. M. (2013). Decision Tree Classifiers for Automated Medical Diagnosis. *Neural Computing & Applications*.

[9] Yang F., Wang H.-Z., Mi H., Lin C.-D., Cai W.-W. (2009). Using Random Forest for Reliable Classification and Cost-Sensitive Learning for Medical Diagnosis. *BMC Bioinformatics*.

[10] Stoean R., Stoean C. (2013). Modeling Medical Decision Making by Support Vector Machines, Explaining by Rules of Evolutionary Algorithms With Feature Selection. *Expert Systems With Applications*.

[11] Liu L. Research on Logistic Regression Algorithm of Breast Cancer Diagnose Data by Machine Learning.

[12] Samadian F., Dalili N., Jamalian A. (2016). Lifestyle Modifications to Prevent and Control Hypertension. *Iranian Journal of Kidney Diseases*.

[13] Charansonney O. L. (2011). Physical Activity and Aging: A Life-Long Story. *Discovery Medicine*.

[14] Cheraghi P., Mihandoost Yeganeh Z., Dosti Irani A., Sangestani M., Cheraghi Z., Khezeli M. (2015). Study on the Prevalence of Hypertension and Its Associated Factors in the Elderly Population. *Journal of Geriatric Nursing*.

[15] Huang Y.-M., Shiyanbola O. O., Smith P. D. (2018). Association of Health Literacy and Medication Self-Efficacy With Medication Adherence and Diabetes Control. *Patient Preference and Adherence*.

[16] Lor M., Koleck T. A., Bakken S., Yoon S., Dunn Navarra A.-M. (2019). Association Between Health Literacy and Medication Adherence Among Hispanics With Hypertension. *Journal of racial and ethnic health disparities*.

[17] Sohrabi M., Karami M., Mirmoeini R. S., Cheraghi Z. (2022). The Relationship Between Health Literacy and Hypertension Control: A Cross-Sectional Study. *The Journal of Tehran Heart Center*.

[18] Ashoorkhani M., Majdzadeh R., Gholami J., Eftekhar H., Bozorgi A. (2018). Understanding Non-Adherence to Treatment in Hypertension: A Qualitative Study. *International Journal of Community Based Nursing and Midwifery*.

[19] Wilkinson R., Garden E., Nanyonga R. C. (2022). Causes of Medication Non-Adherence and the Acceptability of Support Strategies for People With Hypertension in Uganda: A Qualitative Study. *International Journal of Nursing Studies*.

